# Berberine suppresses hepatocellular carcinoma progression by blocking IL-4-JAK1-STAT6-mediated M2 polarization of macrophage

**DOI:** 10.3389/fphar.2025.1734201

**Published:** 2026-01-08

**Authors:** Peng Wang, Yuwen Zhong, Mengkai Li, Qing Liang, Weiyi Jiang, Mengqi Zhuang, Xuecheng Ge, Huixing Li, Yaoshuai Zhang, Yu Qiao, Jiayao Jiang, Heping Hu, Wendi Liu, Feng Qian, Zishu Wang, Lei Sun, Shulong Zhang, Huabang Zhou

**Affiliations:** 1 Department of Hepatobiliary Medicine, Eastern Hepatobiliary Surgery Hospital, Shanghai, China; 2 Shanghai Frontiers Science Center of Drug Target Identification and Delivery, School of Pharmaceutical Sciences, Shanghai Jiao Tong University, Shanghai, China; 3 National Key Laboratory of Innovative Immunotherapy, Shanghai Jiao Tong University, Shanghai, China; 4 Engineering Research Center of Cell & Therapeutic Antibody, Ministry of Education, School of Pharmaceutical Sciences, Shanghai Jiao Tong University, Shanghai, China; 5 Department of Pharmacy, Shanghai Fifth People’s Hospital, Fudan University, Shanghai, China; 6 Anhui Provincial Key Laboratory of Tumor Evolution and Intelligent Diagnosis and Treatment, Department of Medical Oncology, First Affiliated Hospital of Bengbu Medical University, Bengbu Medical University, Bengbu, Anhui, China; 7 Department of General Surgery, Xuhui Central Hospital, Shanghai, China

**Keywords:** berberine, hepatocellular carcinoma, IL-4–JAK1–STAT6 axis, M2 polarization of macrophage, tumor immune microenvironment

## Abstract

Berberine (BBR), an isoquinoline alkaloid extracted from *Coptis chinensis*, is clinically used to treat chronic colitis, diabetes, and other diseases. Although BBR has antitumor effects, it is unclear whether it can inhibit hepatocellular carcinoma (HCC) by modulating the tumor inflammatory microenvironment. In this study, we demonstrated that BBR inhibits HCC development in mice by suppressing the M2 polarization of macrophages. Using an H22 tumor-bearing xenograft mouse model, we found that BBR significantly inhibited H22 tumor growth. Analysis of scRNA-seq results revealed reduced M2 macrophage infiltration and polarization in BBR-treated HCC tissues. Pharmacodynamic studies showed that BBR treatment markedly increased CD8^+^ T cell infiltration and attenuated M2 polarization. *In vitro*, BBR suppressed IL-4 or tumor cell supernatant-induced M2 polarization, as evidenced by decreased expression of M2 polarization marker genes (Arg*1*, *Retnla*, etc.) and reduced JAK1/STAT6 phosphorylation levels. Molecular docking and protein stability assays revealed that BBR directly binds to JAK1’s FERM domain, stabilizing it. Combination therapy with BBR and anti-PD-L1 antibody synergistically inhibited H22 tumor growth. These findings suggest that BBR can reduce the M2 polarization of tumor-associated macrophages (TAMs) by targeting the IL-4-JAK1-STAT6 axis, and combining with anti-PD-L1 antibody may represent a promising therapeutic strategy to enhance BBR’s antitumor efficacy.

## Introduction

Liver cancer remains one of the most prevalent malignancies worldwide, ranking sixth in incidence and serving as a major cause of cancer-related deaths worldwide, accounting for 7.8% of all cancer fatalities. As the third leading cause of cancer-related deaths globally, the incidence and mortality rates of liver cancer continue to rise (Siegel et al., 2025; Bray et al., 2024), primarily comprising hepatocellular carcinoma (HCC) and cholangiocarcinoma (CCA), with HCC accounting for over 80% of cases (Llovet et al., 2021). Current clinical strategies include surgical resection, liver transplantation, transcatheter arterial chemoembolization (TACE), molecular-targeted therapies, and immune checkpoint inhibitors, which have significantly improved patient survival and reduced recurrence rates. These advances highlight the therapeutic potential of targeting either tumor cells or the tumor immune microenvironment (TIME) (Singal et al., 2023; Seyhan et al., 2025). However, mortality rates remain unacceptably high, underscoring the urgent need for innovative treatments to overcome current therapeutic limitations.

A major focus in cancer immunotherapy involves targeting programmed death-ligand 1 (PD-L1). The PD-L1/programmed death-1 (PD-1) axis serves as a critical immune checkpoint pathway that enhances CD8^+^ T cell-mediated tumor killing (Macarulla et al., 2025). However, its efficacy is frequently compromised by immunosuppressive regulation from tumor-associated macrophages (TAMs) within the TIME, which upregulate inhibitory molecules including PD-1, PD-L1, and Tim-3 (Gordon et al., 2017; Dixon et al., 2021). The TIME comprises diverse immune populations, including cytotoxic T cells (CD8^+^ T), dendritic cells (DCs), and macrophages (Christofides et al., 2022). TAMs are one of the most abundant innate immune populations in the TIME, and clinical evidence suggests that high infiltration of TAMs is strongly associated with poor prognosis and immunotherapy resistance in a variety of solid tumors (Pang et al., 2025; Shen et al., 2023). TAMs are highly malleable (Pittet et al., 2022). Signals from the tumor immune microenvironment cause TAMs to exhibit an inhibitory immune response: M2-TAMs. These M2-TAMs secrete various immunosuppressive factors (e.g., Arg1, IL-10) that enhance tumor cell migration and invasion while fostering an immunosuppressive milieu. Additionally, a specialized Angio-TAM subset promotes tumor angiogenesis through vascular endothelial growth factor (VEGF) and platelet-derived growth factor (PDGF) secretion, typically displaying M2-like polarization characteristics (Ma et al., 2022).

The Janus kinase/signal transducer and activator of transcription (JAK-STAT) pathway serves as a crucial mediator for type I and II cytokines. Notably, excessive activation of JAK1 has been identified as a key factor inducing inflammatory disorders in various diseases, including COVID-19 (Hammersen et al., 2023), and systemic lupus erythematosus (Hirahara et al., 2016). There is a relative paucity of studies on the regulatory role of JAK1 in the development of malignant tumors, especially HCC. Interleukin-4 (IL-4) can bind to type I IL-4R intracellularly to activate the JAK1-STAT6 pathway inducing M2 polarization of TAMs (Roskoski, 2016), upregulating checkpoint molecules (such as PD-L1), and recruiting regulatory T cells, thereby synergistically promoting tumor immune escape (Jiménez-Garcia et al., 2015). Growing experimental evidence demonstrates that modulating TAM activity can enhance the efficacy of tumor immunotherapy. However, the specific mechanisms underlying this process in the context of HCC remain unclear. In particular, systematic research on how natural products regulate TAMs to remodel the T and improve responses to immune checkpoint inhibitors (ICIs) is still lacking. Further exploration of potential intervention strategies and therapeutic value is warranted.

Small-molecule compounds extracted from natural products have the advantages of low toxicity, few side effects, and good tolerance, and play a key role in tumor treatment. Berberine (BBR) is a natural isoquinoline alkaloid widely found in Chinese herbs such as *Coptis chinensis*, *Phellodendron amurense*, and *Berberis julianae*, which is a commonly used clinical drug in China. It has multi-target and multi-mechanism pharmacological effects, and is widely used in the treatment of human diseases such as diabetes (Wang et al., 2022), infectious diarrhea (Chen et al., 2015), and the postoperative treatment of gastric cancer (Wang Y. et al., 2024). Its biological effects include antibacterial (Li J. et al., 2023), and anti-inflammatory (Nikpoor et al., 2024), etc. There is substantial evidence that BBR can exert anti-tumor effects through multiple pathways, including inhibiting tumor cell proliferation (Sun et al., 2023), and inhibiting angiogenesis (Li et al., 2018). Moreover, BBR has been reported to have a unique role in the regulation of intrahepatic T lymphocytes and restoration of immune functions (Hu et al., 2024). However, whether BBR can remodel the immune microenvironment by modulating the TIME, especially by affecting the M2 polarization of macrophages to achieve immune microenvironment remodeling, still lacks a systematic explanation. In addition, whether BBR can synergistically intervene in the key immune signaling pathways in HCC in combination with PD-L1 inhibitors needs to be further explored.

In this study, we observed that after BBR treatment, tumor growth in mouse-derived hepatocellular carcinoma cells H22 in loaded mice was significantly inhibited. Single-cell RNA sequencing (scRNA-seq) results showed that M2 polarization of macrophages was reduced in the HCC immune microenvironment. Berberine treatment promoted the infiltration of CD8^+^ T cells and reduced the proportion of CD206^+^ M2-macrophages. By binding to JAK1, BBR inhibited the IL-4-JAK1-STAT6 axis to reduce M2 polarization of TAMs. Moreover, the combination of BBR with anti-PD-L1 antibody synergistically enhanced anti-tumor immunity. In summary, our study reveals a new treatment method for HCC and clarifies its underlying mechanism, providing new ideas for the combination therapy of natural products and immune checkpoint inhibitors.

## Materials and methods

### Mice

Wild-type female Balb/c mice (6–10 weeks old) were obtained from Lingchang (Shanghai, China). All mice were bred and maintained under specific pathogen-free conditions at Shanghai Jiao Tong University (Shanghai, China). Animals were housed under a 12-h light/dark cycle with stable temperature (20 °C–24 °C) and humidity (45%–65%). All animal experimental procedures were conducted in accordance with the “Guidelines for the Management of Laboratory Animals” (2024 Revision) issued by the National Science Commission.

### Data collection

The genomic analysis dataset GSE271243 was downloaded from the Gene Expression Omnibus (GEO) database (http://www.ncbi.nlm.nih.gov/geo). This dataset contained 18 completely resected HCC tissue samples from three groups of HCC mouse models: HC vs. LB vs. HB (HB group: BBR 30 mg/kg; LB group: BBR 10 mg/kg; HC group: blank solvent only, n = 6 per group). From the HC and HB groups, one tissue sample each (GSM8372134 and GSM8372142) was randomly selected for data processing and analysis.

### scRNA-seq data processing

The Seurat (v5.3.0, RRID: SCR_016341) R package was employed for comprehensive analysis of the scRNA-seq data. Highly variable genes within the dataset were selected for Principal Component Analysis (PCA) dimensionality reduction. Significant principal components (PCs) were selected based on the elbow plot, and the Louvain algorithm was applied for cell clustering. Clusters were visualized using Uniform Manifold Approximation and Projection (UMAP) for dimensionality reduction. The Wilcoxon rank-sum test was used to compare gene expression profiles between clusters to identify marker genes for each cluster. Cell types were annotated by referencing the expression of well-established marker genes and by comparison with publicly available single-cell datasets. Differentially expressed genes between clusters of interest were identified using the FindMarkers function.

### Cell culture

Bone marrow-derived macrophages (BMDMs) were isolated from mouse femurs and tibias according to previous studies (Zhang et al., 2024). Briefly, cells were cultured in Dulbecco’s Modified Eagle Medium (DMEM, Gibco, USA) supplemented with 10 ng/mL recombinant mouse M-CSF for 6 days. On day 7, cells were treated with various methods for further assays. The mouse macrophage cell line RAW 264.7 and mouse-derived hepatocellular carcinoma cell line H22 were obtained from the American Type Culture Collection (ATCC, Manassas, VA, USA). RAW 264.7 and H22 cells were cultured in Roswell Park Memorial Institute (RPMI) medium (RPMI-1640, Gibco) or DMEM supplemented with 10% FCS (Gibco/ThermoFisher) and 1% penicillin/streptomycin (Yeasen Biotech), respectively.

### Animal models

H22 cells (5 × 10^5^) in PBS were subcutaneously implanted into the left flank of Balb/c mice. Starting from day 6 post-implantation, tumor length (A) and width (B) were measured every 2 days. The H22 tumor volume was calculated using the formula: Tumor volume=(A × B^2^)/2. The mice were humanely euthanized via cervical dislocation, and the tumors were excised and photographed. The maximal tumor size was in line with the animal experimental protocol approved by the Ethics Committee of Shanghai Jiao Tong University. The tumor weight was measured. For the therapeutic studies, mice were randomized into treatment groups, and tumor-bearing mice received intragastric administration (i.g.) of BBR (molecular formula C_20_H_18_NO_4_^+^, molecular weight 336.4) 5 mg/kg or 10 mg/kg every 3 days starting from day 6 for three doses. For immune checkpoint inhibitor treatment, tumor-bearing mice were administered anti-PD-L1 antibody (10 mg/kg) via intraperitoneal injection (i.p.) every 3 days for three doses. The synergy between BBR and anti-PD-L1 was quantitatively assessed using the Bliss independence model, where the expected additive effect (E_Exp_) was calculated as EExp = E_A_ + E_B_ − (E_A_ × E_B_), with E_A_ and E_B_ representing the tumor growth inhibition rates of BBR and anti-PD-L1 monotherapies, respectively. Bliss score = E_AB_ − E_Exp_.

### Flow cytometry

Tumor tissues were shredded and digested with collagenase D (1.0 mg/mL) and DNase I (50 U/mL) (Sigma-Aldrich, St. Louis, USA). Single-cell suspensions were obtained using a gentleMACS Octo dissociator (Miltenyi Biotec, Bergisch Gladbach, Germany) with the program for tumors. The cells were then incubated with FACS buffer and stained with antibodies. For tumor-associated macrophages (TAMs), the following antibodies were used: Fixable Viability Stain 510 (564,406, BD Pharmingen), APC-Cy7-conjugated CD45 (557,659, BD Pharmingen), PE-Cy7-conjugated Ly6C (560,593, BD Pharmingen), BV650-conjugated Ly6G (740,554, BD Pharmingen), FITC-conjugated CD11b (561,688, BD Pharmingen), BV605-conjugated F4/80 (743,281, BD Pharmingen), AF647-conjugated I-A/I-E (562,367, BD Pharmingen), PE-conjugated CD206 (568,273, BD Pharmingen). For T cells, the following antibodies were used: FITC-conjugated CD3 (553,061, BD Pharmingen), BUV737-conjugated CD4 (612,761, BD Pharmingen), PE-CF594-conjugated CD8 (562,315, BD Pharmingen). All Sample data were collected using the LSRFortessa™ X-20 flow cytometer (BD Biosciences, San Jose, USA), followed by analyses using FlowJo software (Version 10.8.1; BD Life Sciences, RRID: SCR_008520).

### Total cellular RNA was extracted using TRIzol reagent (invitrogen)

cDNA was synthesized using the ReverTra Ace qPCR RT kit (Toyobo). RT-qPCR was performed by mixing primers with SYBR green RT-PCR master mix (Toyobo) on the StepOne Plus system (Thermo Fisher Scientific). Data were normalized using GAPDH as a reference. The primer sequences are shown in the table below.

**Table:** 

​	Foward	Reverse
*Gapdh*	CAT​CAC​TGC​CAC​CCA​GAA​GAC​TG	ATG​CCA​GTG​AGC​TTC​CCG​TTC​AG
Arg*1*	CAT​TGG​CTT​GCG​AGA​CGT​AGA​C	GCT​GAA​GGT​CTC​TTC​CAT​CAC​C
*Retnla*	CAA​GGA​ACT​TCT​TGC​CAA​TCC​AG	CCA​AGA​TCC​ACA​GGC​AAA​GCC​A
*Il10*	CGG​GAA​GAC​AAT​AAC​TGC​ACC​C	CGG​TTA​GCA​GTA​TGT​TGT​CCA​GC
*Mrc1*	GTT​CAC​CTG​GAG​TGA​TGG​TTC​TC	AGG​ACA​TGC​CAG​GGT​CAC​CTT​T
*Tgfb1*	TGA​TAC​GCC​TGA​GTG​GCT​GTC​T	CAC​AAG​AGC​AGT​GAG​CGC​TGA​A
*Chil3*	TAC​TCA​CTT​CCA​CAG​GAG​CAG​G	CTC​CAG​TGT​AGC​CAT​CCT​TAG​G

### Western blotting

BMDM cells were seeded in 6-well plates and pretreated with BBR (0, 3, 10, 30 μM) for 1 h. Cells were then stimulated with H22 tumor cell-conditioned medium or IL-4 (20 ng/mL) for 1 h. BMDM cells were lysed in RIPA buffer (Sangon) containing protease inhibitors (Sigma-Aldrich). Lysates were centrifuged at 4 °C, and supernatants were collected as protein extracts. Protein concentrations were measured using a BCA kit (Beyotime). Samples were separated on 8% or 10% acrylamide gels by electrophoresis at 120 V. Proteins were transferred to nitrocellulose membranes (GE Healthcare, Little Chalfont, UK) and blocked with 5% skim milk for 1.5 h to prevent nonspecific binding. Primary antibodies were incubated overnight at 4 °C, including: p-STAT6 (56554S, CST), t-STAT6 (9362S, CST), p-JAK1 (3331S, CST), t-JAK1 (3344S, CST), β-actin (8457S, CST), Arg-1 (89872SF, CST), Retnla (Abcam, AB39626). Secondary antibodies were incubated for 2 h. After washing, proteins were visualized using ECL detection reagent (ThermoFisher Scientific, Waltham, USA) and a ChemiDoc XRS + system (Bio-Rad, Hercules, USA).

### Co-immunoprecipitation assay

BMDM cells were seeded in 10-cm dishes and pretreated with BBR (30 μM) for 1 h, followed by stimulation with IL-4 (20 ng/mL) for 30 min. Control cells were treated with vehicle alone or IL-4 only. After treatment, cells were washed twice with PBS and lysed in in mild lysis buffer (NP-40 lysis buffer) supplemented with protease and phosphatase inhibitors. Lysates were cleared by centrifugation at 20,000 × g for 20 min at 4 °C. For each sample, 50 µL of supernatant was mixed with 50 µL of 2×loading buffer as input control. The remaining supernatant was equally divided for incubation with either 2 µg of mouse IgG or 2 µg of anti-JAK1 antibody (3344S, CST) overnight at 4 °C. Protein A/G magnetic beads were then added and incubated for 2 h. Beads were washed four times with NP-40 lysis buffer, and bound proteins were eluted in loading buffer for subsequent Western blot analysis.

### Molecular docking

The mouse JAK1 structure (PDB code: 7T6F) was modeled by Glassman (Stanford University School of Medicine, USA). The mouse STAT6 structure (PDB code: 4Y5U) was modeled by Jing Li (University of Chinese Academy of Sciences, China). BBR was drawn using ChemDraw Professional (v22.0.0.22; PerkinElmer, RRID: SCR_016768). JAK1 and STAT6 were preprocessed with PyMOL (v2.6; Schrödinger, RRID: SCR_000305), then docked with BBR using AutoDock (v1.5.7; RRID: SCR_012746). Docking results were analyzed and visualized with PyMOL. Interaction diagrams were generated using LigPlot+ (v2.2.5; EMBL-EBI, RRID: SCR_018290) to illustrate hydrogen bonds, hydrophobic interactions, and other critical contacts for binding mode analysis.

### Cellular thermal shift assay (CETSA)

For the intact cell assay, RAW 264.7 cells were treated with BBR (30 μM) for 1 h, harvested, and washed twice with PBS to remove unbound molecules. The supernatant was removed, and cells were resuspended in PBS containing protease inhibitors. The cell suspension was evenly distributed into 0.2 mL PCR tubes and heated at a temperature gradient for 3 min. After rapid freeze-thaw cycles in liquid nitrogen (three repetitions), cells were centrifuged at 20,000 × g for 20 min, and the supernatant was collected. A loading buffer was added for subsequent Western blot analysis.

For the cell lysate assay, after washing with PBS, cells were resuspended in PBS containing protease inhibitors and subjected to three rapid freeze-thaw cycles in liquid nitrogen. The lysate was centrifuged at 4 °C and 20,000 × g for 20 min to remove debris. The supernatant was collected, treated with BBR (30 μM) at room temperature for 30 min, and then denatured at a temperature gradient for 3 min. A loading buffer was added for subsequent Western blot analysis.

### Drug affinity responsive target stability (DARTS)

Cells were washed twice with PBS and lysed in mild lysis buffer (NP-40 lysis buffer) containing 100× protease inhibitor cocktail (PIC) on ice for 10 min. Cells were scraped and centrifuged at 4 °C and 18,000 × g for 10 min to obtain the protein supernatant. The supernatant was divided into separate tubes and incubated with DMSO or various concentrations of BBR (0, 3, 10, 30 μM) at room temperature for 1 h. Samples were then digested with Pronase (Roche) (enzyme-to-protein ratio = 1:800) at room temperature for 30 min. The digestion reaction was stopped by adding 100× protease inhibitor PMSF, followed by incubation on ice for 10 min. A loading buffer was added for subsequent Western blot analysis.

### Statistical analysis

Data are presented as the mean ± SEM, with a sample size of n ≥ 3 for all experiments. Detailed statistical parameters of the analyses are provided in Figure Legends. For two groups, data were compared using a two-tailed Student’s test. For multiple groups, data were compared using one-way analysis of variance (ANOVA) Dunn’s *post hoc* analysis or two-way ANOVA (Two-way ANOVA) Tukey’s *post hoc* analysis. Significance is presented as *P < 0.05, **P < 0.01, and ***P < 0.001.

## Results

### BBR inhibits H22 tumor growth

**FIGURE 1:**
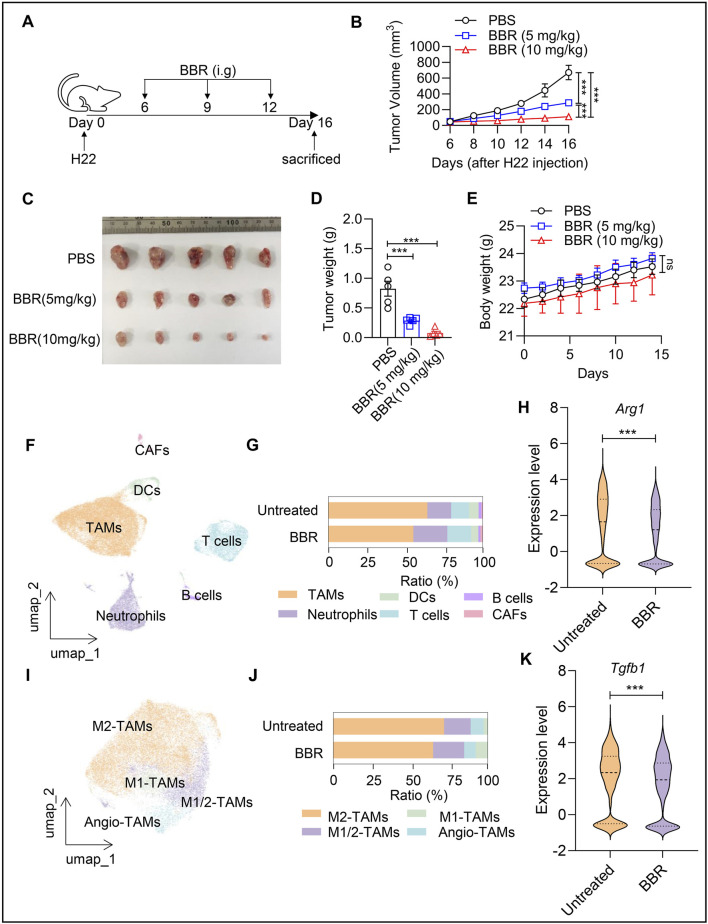
Berberine inhibits H22 tumor growth *in vivo*. **(A)** Schema of the mouse tumor model: Female Bal/bc mice engrafted with H22 tumor cells (5 × 10^5^) received intragastric (i.g.) BBR (0, 5, 10 mg/kg) challenge. Tumors were removed and analyzed on day 16. **(B)** Tumor volume was measured in mice every 2 days from day 6 after tumor implantation. (n = 5). **(C–E)** On day 16 after tumor cell implantation, the tumors in mice were removed and weighed. (n = 5). Tumor growth curves **(C)**, tumor weight **(D)**, and mean body weight **(E)** are shown. **(F)** UMAP plot shows the cluster analysis of all samples, with each color representing a distinct cell type. **(G)** Bar plot illustrates proportional differences in cells between BBR-treated and untreated mice. **(H)** Umap plot displays four types of macrophages. **(I)** Dot plot presents the expression of signature genes in four types of macrophages. **(J,K)** Expression of Arg*1* **(i)** and *Tgfb1* **(j)** in TAMs from BBR-treated and untreated mice. Data (mean ± SEM) are representative of three independent experiments. *P < 0.05, **P < 0.01, ***P < 0.001.

To examine whether BBR has an inhibitory effect on HCC, we subcutaneously implanted mouse-derived hepatocellular carcinoma cells H22 into the left flank of Balb/c mice. The BBR treatment protocol is shown in Figure 1A. We found that both 5 mg/kg and 10 mg/kg BBR effectively inhibited tumor growth, and the inhibitory effect was positively correlated with BBR dosage (Figures 1B–D). At 16 days after H22 cell inoculation in Balb/c mice, tumor volume in the PBS group reached 600 mm^3^. The final tumor volume in the BBR (10 mg/kg) group was less than 200 mm^3^. BBR treatment significantly reduced tumor volume by more than threefold (Figure 1C). Tumor weight in the BBR (10 mg/kg) group decreased by at least 50% compared to the PBS group (Figure 1D). Notably, BBR administration showed no significant impact on mouse body weight (Figure 1E), confirming its safety profile. These data unequivocally underscore the potent anti-tumor effects of BBR in HCC.

To investigate the impact of BBR treatment on the tumor immune microenvironment in HCC, we analyzed single-cell data from BBR-treated and untreated mouse HCC tissues in the GSE271243 dataset (one randomly selected sample from each group). Unsupervised clustering of the single-cell data identified six distinct cell populations, including TAMs, neutrophils, DCs, T cells, cancer-associated fibroblasts (CAFs), and B cells (Figure 1F). Notably, the proportion of TAMs was significantly reduced in BBR-treated mice. In contrast, the proportions of T cells and neutrophils were higher in BBR-treated mice compared to untreated mice (Figure 1G). Further analysis of macrophage subsets via scRNA-seq revealed four types: M1-TAMs (characterized by high expression of *Ptgs2*, *Tnf*, and cytokines *Il1b*, *Ccl2*, *Osm*), M2-TAMs (characterized by high expression of Arg*1*, *Mrc1*, *Chil3*, and *Tgfb1*), M1/M2 Transitional TAMs (M1/M2-TAMs, characterized by co-expression of these genes), and Angio-TAMs (characterized by high expression of *Col1a1*) (Figure 1H). Among these subsets, the proportion of M2-TAMs was notably reduced in BBR-treated mice. Whereas the proportion of M1-TAMs was slightly elevated, the difference in the number of cells was not significant, suggesting that the reduced macrophage population was predominantly M2-TAMs (Figure 1I). Differential gene expression analysis using FindMarkers on tumor-associated macrophages revealed significant downregulation of M2 polarization markers (Arg*1*, *Tgfb1)* post-BBR treatment (Figures 1J,K). These data suggest that BBR treatment alters the composition of the tumor microenvironment in HCC mice, particularly affecting immune cell infiltration.

### BBR promotes immune cell infiltration in tumor immune microenvironment

**FIGURE 2:**
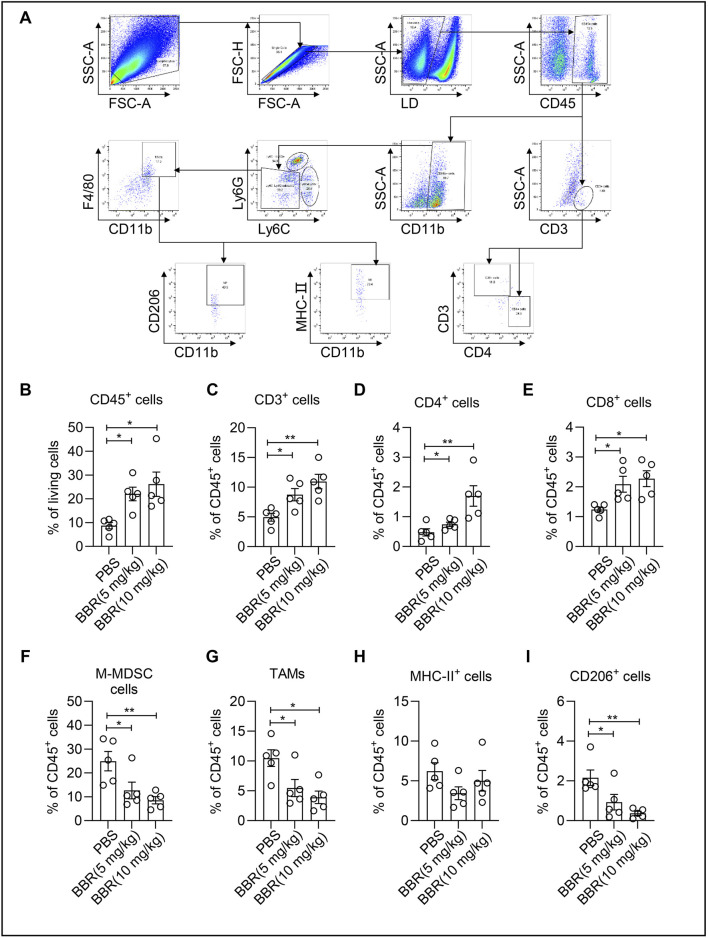
Berberine promotes anti-tumor immunity *in vivo*. **(A)** Flow cytometry analysis of immune cells in the BBR-treated mouse H22 tumor tissues. **(B–I)** Flow cytometry analysis for the percentages of CD45^+^ lymphocytes in tumor tissues **(B)**, Percentage of CD3^+^ cells within the gated CD45^+^ cells in melanoma tissues **(C)**, CD3^+^CD4^+^ T cells **(D)**, CD3^+^CD8^+^ T cells **(E)**, CD45^+^CD11b^+^Ly6G^−^Ly6C^+^ M-MDSC **(F)**, CD45^+^CD11b^+^Ly6G^−^LY6C^−^F4/80^+^ TAMs **(G)**, CD11b^+^F4/80^+^MHC-Ⅱ^+^ macrophages **(H)**, and CD11b^+^F4/80^+^CD206^+^ macrophages **(I)**, within CD45^+^ population in tumors. Data (mean ± SEM) are representative of three independent experiments. *P < 0.05; **P < 0.01; ***P < 0.001.

To further investigate how BBR modulates immune cells in the tumor microenvironment, we analyzed immune cell populations of mouse-derived hepatocellular carcinoma cells H22 tumor-bearing xenograft mice given BBR treatment by flow cytometry (Figure 2A). First, we found that BBR treatment increased the proportion of CD45^+^ immune cells among live cells, with the low-dose group reaching approximately 20% of total cells (Figure 2B). Moreover, BBR promoted increased proportions of CD3^+^ T cells, CD4^+^ T cells and CD8^+^ T cells, with the high-dose group showing more than 2-fold increases (Figures 2C–E). Simultaneously, BBR treatment reduced the proportion of monocytic myeloid-derived suppressor cells (M-MDSCs) (Figure 2F). Compared to the PBS group, BBR treatment decreased the proportion of TAMs in immune cells, with the low-dose group showing a significant 50% reduction (Figure 2G). While no significant effect was observed on MHC-II^+^ M1-macrophages (Figure 2H), BBR treatment markedly reduced the proportion of CD206^+^ M2-macrophages (Figure 2I). These findings suggest that BBR treatment can enhance CD8^+^ T cell infiltration and reduce M2 polarization of tumor-infiltrating macrophages, thereby promoting anti-tumor immunity *in vivo*.

### BBR inhibits H22 tumor cell-conditioned medium and IL-4-induced M2 polarization of macrophages

**FIGURE 3:**
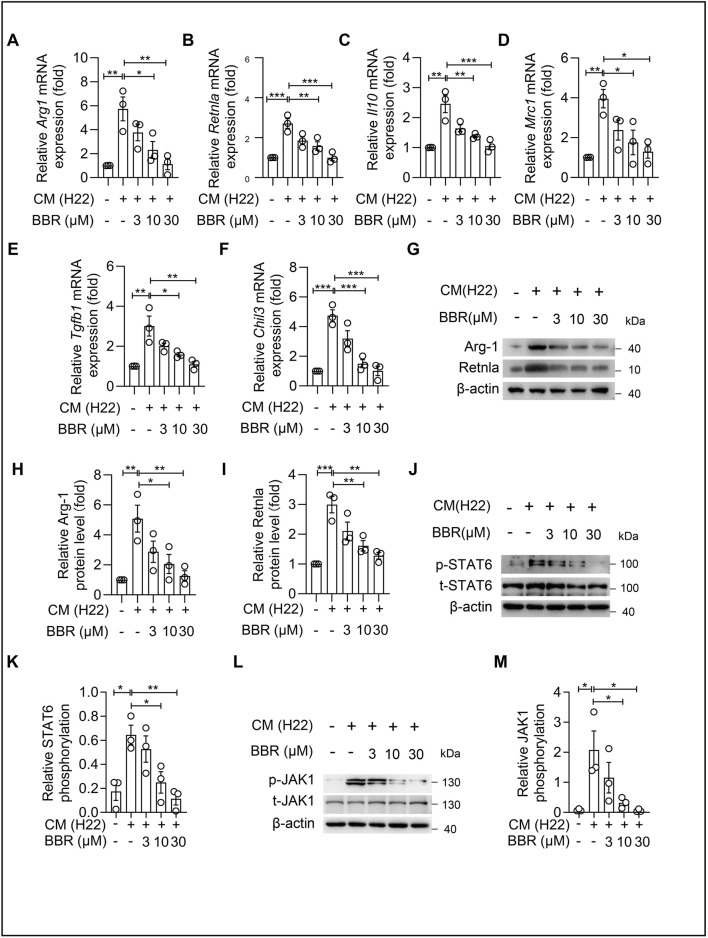
Berberine inhibits the activation of the JAK-STAT6 signal pathway induced by H22 conditional medium. **(A–F)** Relative transcription levels of M2-type signature genes in BMDM, including Arg*1* **(A)**, *Retnla* **(B)**, *Il10* **(C)**, *Mrc1* **(D)**, *Tgfb1* **(E)**, and *Chil3* **(F)**. BMDM were treated with H22 conditional medium for 4 h. **(G)** Immunoblot analysis of Arg-1 and Retnla protein expression in BMDMs. **(H,I)** Quantitative analysis of **(H)** Arg-1 and **(I)** Retnla protein levels normalized to β-actin. **(J)** Immunoblot detection of p-STAT6 protein expression in BMDM. **(K)** Quantitative statistics of p-STAT6/STAT6 are shown. **(L)** Immunoblot detection of p-JAK1 protein expression in BMDM. **(M)** Quantitative statistics of p-JAK1/JAK1 are shown. Data (mean ± SEM) are representative of three independent experiments. *P < 0.05; **P < 0.01; ***P < 0.001.

To investigate whether BBR can inhibit M2 polarization of macrophages *in vitro*, we co-cultured BMDM cells with H22 tumor cell-conditioned medium. We found that H22 tumor cell-conditioned medium promoted the expression of M2 macrophage marker genes, while BBR significantly suppressed these genes at the mRNA level. The mRNA levels of Arg*1*, *Retnla*, *Il10*, *Mrc1*, *Tgfb1*, and *Chil3* were all inhibited by BBR (Figures 3A–F), with Arg*1* expression being suppressed by up to 3-fold. Consistently, at the protein level, BBR markedly suppressed tumor-conditioned medium-induced upregulation of Arg-1 and Retnla (Figures 3G–I). In addition, at the protein level, BBR inhibited tumor-conditioned medium-induced phosphorylation of STAT6 (Figures 3J,K), and administration of 30 μm BBR resulted in a 30% decrease in the phosphorylation status of STAT6 compared to the PBS group. Also, a certain concentration of BBR inhibited the phosphorylation activation of JAK1 (Figures 3L,M). These results suggest BBR inhibits H22 tumor cell-conditioned medium-induced M2 polarization of macrophages through the JAK1/STAT6 pathway.

**FIGURE 4:**
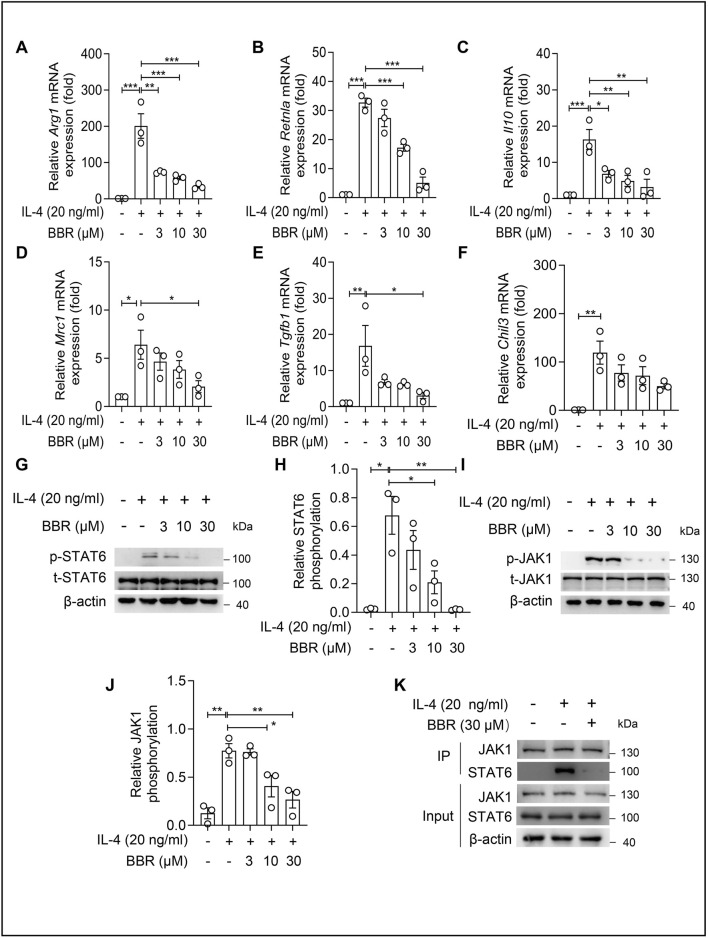
Berberine inhibits the activation of the JAK-STAT6 signal pathway induced by IL-4. **(A–F)** Relative transcription levels of M2-type signature genes in BMDMs stimulated by IL-4 (20 ng/mL) for 4 h. Significant changes were observed in Arg1 **(A)**, Retnla **(B),** Il10 **(C)**, Mrc1 **(D)**, Tgfb1 **(E)**, and Chil3 **(F)**. **(G,H)** Western blotting analysis of p-STAT6 protein expression in BMDMs. Representative immunoblots **(G)** and quantitative analysis of p-STAT6/STAT6 **(H)** are shown. **(I,J)** Content analysis of p-JAK1 protein expression in BMDMs. Representative immunoblots **(I)** and quantitative analysis of p-JAK1/JAK1 **(J)** are shown. **(K)** Co-immunoprecipitation analysis of JAK1-STAT6 interaction in BMDMs under IL-4 (20 ng/mL) stimulation with or without BBR (30 μM) treatment. Data (mean ± SEM) are representative of three independent experiments. *P < 0.05; **P < 0.01; ***P < 0.001.

Since IL-4 can induce macrophage differentiation into the M2 phenotype by activating the JAK1/STAT6 pathway, we examined whether BBR could inhibit IL-4-induced macrophage polarization. We found that IL-4 treatment significantly upregulated the mRNA expression levels of M2-type macrophage marker genes. BBR treatment dose-dependently inhibited the mRNA lev els of Arg*1*, *Retnla*, *Il10*, *Mrc1*, *Tgfb1*, and *Chil3* (Figures 4A–F). Furthermore, 10 μM BBR significantly inhibited IL-4-induced phosphorylation of STAT6 (Figures 4G,H) and JAK1 (Figures 4I,J). Furthermore, co-immunoprecipitation assays revealed that BBR substantially disrupted the physical interaction between JAK1 and STAT6 induced by IL-4 stimulation (Figure 4K), providing mechanistic insight into how BBR attenuates JAK1-STAT6 signaling. These findings indicate that BBR reduces M2 polarization of macrophages by inhibiting the IL-4-JAK1-STAT6 axis.

### BBR binds to and inhibits JAK1

**FIGURE 5:**
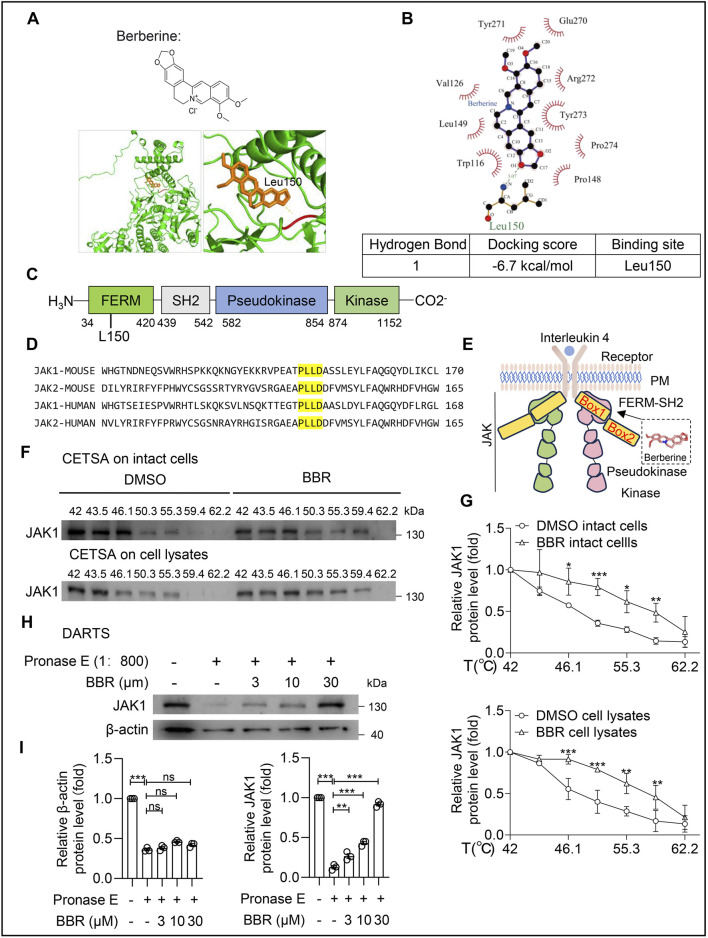
JAK1 is identified as a novel target for Berberine. **(A)** Chemical structure of BBR and schematic diagram illustrating the interaction between JAK1 and BBR molecules. **(B)** Ligplot analysis of the interacting amino acid residues between JAK1 and BBR, Binding energies calculated by AutoDock Vina. **(C)** Diagram of JAK1 domains with corresponding amino acid positions labeled. **(D)** Protein sequence alignment of JAK1 and JAK2 between human and mouse. Partial amino acid sequences of JAK binding to BBR were aligned using SnapGene. The conserved sites are shown in yellow. **(E)** Structure of JAK1 protein. **(F)** CETSA analysis of JAK1 thermal stability in both intact cells and lysates of RAW264.7 cells treated with BBR (30 μM), assessed across a temperature gradient (42.0 °C–62.2 °C) **(G)** Quantitative statistics of JAK1 in **(F)** are shown. **(H)** Cell lysis was incubated with BBR (0–30 μM), followed by Pronase digestion. Immunoblot detection of JAK1 protein expression in RAW264.7. **(I)** Quantitative statistics of JAK1in **(H)** are shown. Data (mean ± SEM) are representative of three independent experiments. *P < 0.05; **P < 0.01; ***P < 0.001.

Given that BBR can disrupt the JAK1-STAT6 interaction and inhibits phosphorylation events along the IL-4-JAK1-STAT6 signaling axis, we hypothesized that BBR might directly bind to JAK1 and inhibit its activity. Through structure-based simulated docking, we used PyMOL software to perform molecular docking verification of active compounds against core targets and constructed a specific binding model (Figure 5A). Additionally, Ligplot was used to identify hydrogen bond and binding sites between them, with detailed binding energy (−6.7 kcal/mol) listed (Figure 5B). Specifically, BBR binds to JAK1 protein by forming a hydrogen bond with residue Leu150 in the FERM domain (Figure 5C), and the key amino acid residues involved in docking are highly conserved among different species of JAK1/2 (Figure 5D). The FERM domain is primarily responsible for the binding between JAK kinase and the intracellular Box1 portion of cytokine receptors, and the interaction between JAK1 and cytokine receptors is crucial for signal activation (Haan et al., 2006). Therefore, BBR’s direct binding to the FERM domain of JAK1 may impair JAK1-cytokine receptor interaction (Figure 5E).

Cellular thermal shift assay (CETSA) results showed that in intact RAW 264.7, at 50.3 °C, JAK1 protein in the DMSO-treated group had degraded by over 50% compared to low temperature. In comparison, the BBR-treated group required 59.4 °C to reach the same degradation. Moreover, in both intact cells and cell lysates, JAK1 protein in the DMSO-treated group was completely degraded at 59.4 °C, whereas the BBR-treated group required 62.2 °C for complete degradation. These results indicate that BBR enhances the thermal stability of JAK1 protein in both intact cells and cell lysates (Figures 5F,G). Correspondingly, the drug affinity responsive target stability (DARTS) assay showed that in the presence of BBR, the proteolytic capacity of protease against JAK1 protein was also weakened (Figures 5H,I). As BBR concentration increased, the anti-proteolytic ability of JAK1 protein gradually improved, suggesting that JAK1 protein is a potential direct molecular target for BBR’s anti-tumor activity.

### Combination therapy of BBR and anti-PD-L1 antibody enhances tumor suppression

**FIGURE 6:**
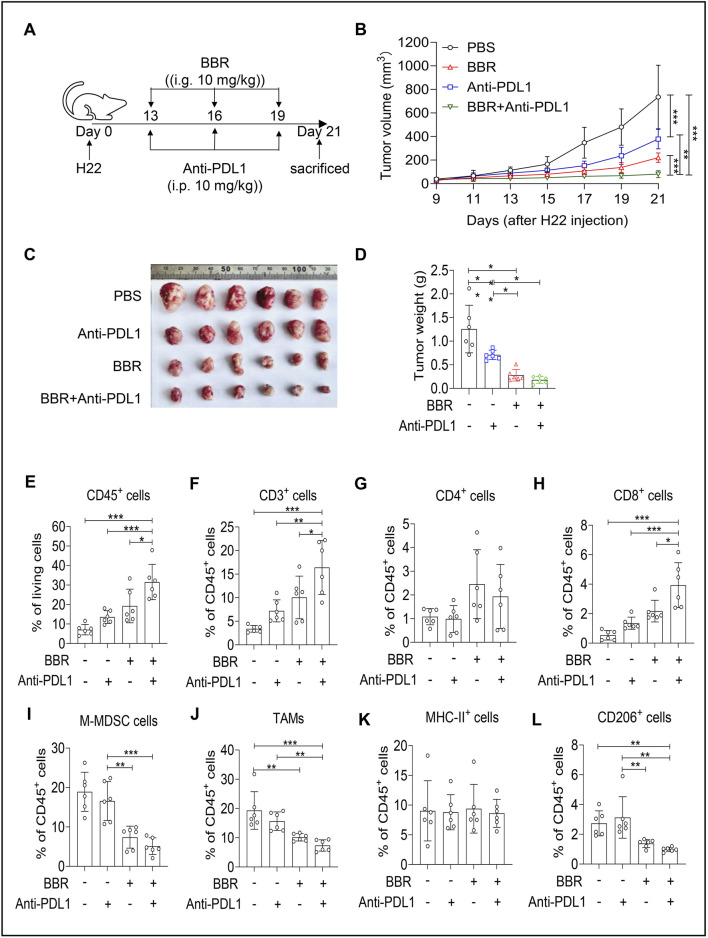
Synergistic therapeutic effects of Berberine and Anti-PDL1 antibody in treating H22 tumor. **(A)** Schema of the mouse tumor model: Female Bal/bc mice were engrafted with H22 tumor cells (5 × 10^5^), received intragastric (i.g.) BBR (10 mg/kg), intraperitoneal (i.p.) Anti-PD-L1 antibody (10 mg/kg), or combination treatment. Tumors were removed and analyzed on day 21. **(B)** Tumor volume was measured every 2 days from day 9 after tumor implantation (n = 6). **(C,D)** On day 21 after tumor cell implantation, the tumors in mice were removed and weighed. (n = 6). Tumor growth curves **(C)** and tumor weight **(D)** are shown. **(E–L)** Flow cytometry analysis for the percentages of CD45^+^ lymphocytes in tumor tissues **(E)**, Percentage of CD3^+^ cells within the gated CD45^+^ cells in melanoma tissues **(F)**, CD3^+^CD4^+^ T cells **(G)**, CD3^+^CD8^+^ T cells **(H)**, CD45^+^CD11b^+^Ly6G^−^Ly6C^+^ M-MDSC **(I)**, CD45^+^CD11b^+^Ly6G^−^LY6C^−^F4/80^+^ TAMs **(J)**, CD11b^+^F4/80^+^MHC-Ⅱ^+^ macrophages **(K)**, and CD11b^+^F4/80^+^CD206^+^ macrophages **(L)**, within CD45^+^ population in tumors. Data (mean ± SEM) are representative of three independent experiments. *P < 0.05; **P < 0.01; ***P < 0.001. Schematic diagram illustrating the role of BBR in inhibiting tumor progression. BBR suppresses M2 polarization of tumor-associated macrophages in the tumor microenvironment and enhances the tumor-killing capacity of cytotoxic T lymphocytes, thereby inhibiting tumor progression. Mechanistically, BBR binds to the JAK1 protein in macrophages, inhibiting IL-4-induced phosphorylation of the JAK1-STAT6 signaling axis. This inhibition reduces the secretion of alternative activation genes by macrophages and blocks their pro-tumorigenic functions.

To investigate the potential synergistic effects of combining BBR with anti-PD-L1 antibody in treating HCC in mice, we administered anti-PD-L1 antibody alongside BBR to H22-bearing mice (Figure 6A). Results showed that both anti-PD-L1 antibody and BBR monotherapies exhibited anti-H22 solid tumor effects *in vivo*, with BBR monotherapy demonstrating superior efficacy compared to anti-PD-L1 antibody monotherapy. Moreover, the combination of these two therapies was more effective in inhibiting tumor growth than either BBR or anti-PD-L1 antibody monotherapy alone (Figure 6B), as quantitatively demonstrated by a calculated Bliss synergy score of +0.0045, indicating synergistic interaction. Compared to BBR monotherapy and anti-PD-L1 antibody monotherapy groups, H22 tumor-bearing mice receiving combination therapy showed significantly reduced solid tumor volume and weight (Figures 6C,D). We further analyzed immune cell infiltration in tumor tissues. As shown in Figures 6E–L, compared to PBS group, the combination therapy group showed significant increase in CD45^+^ immune cells, accounting for approximately 30% of total cells (Figure 6E). The tumor-suppressive T cell populations also markedly expanded, with CD3^+^ T cells increasing by over 10% (Figure 6F). Additionally, the proportion of CD4^+^ T cells among CD45^+^ T cells increased (Figure 6G). Notably, CD8^+^ T cell numbers increased approximately 3-fold compared to PBS group (Figure 6H). The combination of anti-PD-L1 antibody and BBR further reduced M-MDSC cell numbers (Figure 6I) and decreased the proportion of TAMs (Figure 6J). Specifically, MHC-II^+^ anti-tumor M1-macrophages showed minimal changes (Figure 6K), while tumor-promoting CD206^+^ M2-macrophages were significantly reduced after combination treatment (Figure 6L). These results indicated that the combination therapy enhanced the efficacy of anti-PD-L1 antibody therapy in HCC treatment.

## Discussion

Berberine is a multi-target natural drug with high safety and broad pharmacological activities, clinically used primarily for treating type 2 diabetes while also demonstrating auxiliary effects in cardiovascular diseases, such as lowering blood pressure and improving vascular dysfunction (Meng et al., 2024). Additionally, BBR exhibits anti-tumor effects by inhibiting tumor cell proliferation, metastasis, and inducing apoptosis (Wang Z. et al., 2024). BBR binds to the intracellular domain of transforming growth factor beta receptor 1 (TGFBR1) in pancreatic cancer, protecting endothelial barrier function from disruption induced by TGF-β1-overexpressing tumor cells and mitigating lung metastasis (Tian et al., 2022). Leveraging its lipophilic cationic properties, BBR also targets cancer cell mitochondria (Steinberg et al., 2020), inducing mitochondrial apoptosis to exert anti-cancer effects (Son et al., 2019). However, whether BBR can act as an immunomodulator to regulate the tumor immune microenvironment and suppress HCC progression remains unclear. In this study, we found that BBR inhibited the growth of mouse-derived hepatocellular carcinoma cells H22 tumor. Further analyses showed a significant decrease in the number of TAMs in the TIME and a decrease in the polarization of M2-macrophages in the HCC tissues after BBR treatment, accompanied by an increase in the infiltration of CD8^+^ T cells. These findings suggest BBR may exert anti-tumor effects by modulating immune cell differentiation and function within the tumor microenvironment. Although our study focuses on the key role of macrophage polarization, the transcriptomic data suggest that BBR may exert regulatory effects on neutrophil populations. Future research could prioritize investigating the composition and function of neutrophil subsets in this context.

Subsequent experiments demonstrated that BBR targets JAK1 protein, inhibiting the IL-4-JAK1-STAT6 axis to suppress M2 polarization of tumor-infiltrating macrophages, thereby enhancing tumor suppression. Combination therapy with BBR and anti-PD-L1 antibody showed synergistic therapeutic effects. Collectively, our results indicate that BBR, either alone or combined with anti-PD-L1 antibody, enhances anti-tumor immunity and effectively inhibits H22 tumor progression, offering a novel therapeutic strategy for HCC and guiding future clinical applications of BBR.

The polarization of TAMs is dynamically regulated by various cytokines in the tumor immune microenvironment, and targeted intervention of TAMs polarization represents a highly promising therapeutic strategy in current tumor immunotherapy (Zhang et al., 2020). Multiple natural products can specifically regulate TAMs polarization through different mechanisms to exert anti-tumor effects. For instance, vinblastine (VBL) can promote TAMs polarization toward the M1 phenotype with anti-tumor immune function by inducing NF-κB activation and cytochrome b-245 alpha (Cyba) chain-dependent reactive oxygen species generation; meanwhile, VBL also facilitates nuclear translocation of transcription factor EB (TFEB), significantly enhancing macrophage phagocytic activity (Wang et al., 2023). Current therapeutic strategies targeting TAMs mainly focus on restricting the infiltration of macrophage precursors and reducing the existing M2-TAM population in the TIME. For example, piceatannol (PIC) not only limits M2 polarization of macrophages but also specifically disrupts the TGF-β1-mediated positive feedback loop between M2 macrophages and tumor cells, thereby inhibiting tumor cell invasion and metastatic potential (Chiou et al., 2022). As we continue to explore the complex interactions between BBR and cellular pathways, we discovered that BBR can inhibit M2 polarization of TAMs by modulating IL-4-mediated signaling downstream of macrophages in the TIME. In conjunction with the existing literature reporting that BBR activates CD8^+^ T cells and promotes central memory T cell (Tcm) formation through the coordination of AMPK and JAK3-STAT5 signaling pathways (Li M. et al., 2023), we speculated that BBR may suppress HCC progression through a dual mechanism. Specially, on one hand, it improves the tumor immunosuppressive microenvironment by blocking M2 macrophage formation; on the other hand, it further enhances T cell-mediated tumor killing by relieving M2 macrophage-mediated suppression of CD8^+^ T cells. Given BBR’s inhibitory effects in various tumors, further research into the detailed molecular pathways underlying its mechanisms of action on different cell types, along with exploration of BBR’s anti-tumor properties to develop more effective cancer immunomodulatory strategies, will be crucial in this research field.

There exists a bidirectional interaction between inflammation and tumor microenvironment formation, where persistent inflammatory stimulation under cancerous conditions progressively skews macrophage differentiation toward the immunosuppressive M2-like phenotype (Zamarron and Chen, 2011). The JAK/STAT signaling pathway is highly activated in various human malignancies (Perner et al., 2025). In breast cancer and non-small cell lung cancer, targeting STAT6 or using STAT6 inhibitors can enhance radiosensitivity and reduce M2 polarization of macrophages (He et al., 2023; Nie et al., 2023). Clinical pathological analyses reveal that approximately 54% of non-small cell lung cancers exhibit high STAT6 expression levels, with this abnormal expression pattern being particularly prominent in immune cell populations infiltrating the pulmonary interstitium (Pastuszak-Lewandoska et al., 2017). Starting with ruxolitinib, the first JAK inhibitor approved by the FDA (Liu et al., 2017), targeted therapy against the JAK-STAT signaling pathway has entered a rapid development phase. Currently, new-generation JAK inhibitors, including fedratinib (Harri et al., 2024), Ruxolitinib (Verstovsek et al., 2012) and pacritinib (Cafardi et al., 2022), have successively entered clinical research, with their safety and efficacy data fully validating the clinical value of this pathway as a therapeutic target for tumors. Against this background, we found that BBR can directly bind to JAK1 protein through molecular structure-based simulated docking and protein stability assay results, which in turn prevented JAK1 binding to STAT6 and inhibited M2 polarization of macrophages induced by the JAK1-STAT6 signaling pathway; therefore, the main reason for the therapeutic effect of BBR on tumors is likely to be the direct targeting of the JAK1- STAT6 signaling pathway. Consequently, the primary mechanism for the therapeutic effect of BBR on tumors likely involves direct targeting of the JAK1-STAT6 signaling pathway. These findings suggest that BBR may serve as a JAK1 inhibitor targeting TAMs to remodel the TIME, providing crucial evidence for developing natural product-derived JAK1-targeted inhibitors for HCC treatment.

PD-L1 is highly expressed in tumor cells and antigen-presenting cells within the tumor microenvironment, making PD-L1 immune checkpoint inhibitors (ICIs) crucial for cancer treatment (Liu et al., 2017). However, the clinical efficacy of ICIs often falls short of optimal levels. Combination therapy strategies employing multi-target interventions can effectively address tumor heterogeneity and adaptability while reducing the development of drug resistance (Yi et al., 2022). Monotherapy with vascular endothelial growth factor receptor 2 (VEGFR2) inhibitors shows limited efficacy against glioblastoma multiforme (GBM) in clinical practice, but anti-VEGFR2 therapy can enhance tumor sensitivity to PD-L1 inhibitors by downregulating p21-activated kinase 4 (PAK4) and increasing cytotoxic CD8^+^ T cell infiltration and activation, providing a novel strategy for combined immunotherapy of GBM (Yao et al., 2025). Based on our previous experimental results demonstrating that BBR treatment reduces M2 polarization of macrophages while increasing T lymphocyte infiltration in the TIME, and considering the limited efficacy of PD-L1 inhibitor monotherapy in HCC (Zhang et al., 2025), BBR may complement anti-PD-L1 antibody-mediated ICI blockade. Studies indicate that BBR treatment can modulate T lymphocyte heterogeneity in HCC, leading to overall improvement in T lymphocyte effector functions, specifically decreased exhausted T (Tex) cells and increased functionally activated T cells (Hu et al., 2024). However, Qin et al. (Qin et al., 2010) suggested that BBR may impair antigen-presenting cell function by down-regulating the co-stimulatory molecules CD80 and CD86 as well as cytokines IL-6 and IL-12. Failure to recognize antigens released by tumor cells or problems with antigen processing and presentation can disrupt the anti-tumor response and lead to therapeutic failure of ICIs (Jhunjhunwala et al., 2021), providing a conflicting theoretical rationale for the association of BBR with anti-PD-L1 antibodies. We found a significant reduction in the volume and weight of H22 tumors after combining the anti-PD-L1 antibody with BBR, and we hypothesized that BBR’s lifting of TAMs-mediated immunosuppression might be crucial for its enhanced effect of the anti-PD-L1 antibody. Subsequent experiments confirm this, showing markedly increased CD8^+^ T cells and reduced CD206^+^ M2-macrophages in the combination group compared to monotherapy controls. The study suggests that BBR may overcome anti-PD-L1 antibody monotherapy resistance by down-regulating M2 macrophage marker expression in the TIME, further enhancing cytotoxic CD8^+^ T cell function, thereby promoting antitumor immunity and inhibiting tumor growth. Collectively, our findings highlight the potential of combining natural product BBR with other immunotherapies, providing a clinically translatable new strategy for HCC combination immunotherapy, particularly for anti-PD-L1 antibody treatment-resistant patient populations.

In conclusion, based on the findings of this study, we propose incorporating BBR as a novel biological agent into future clinical treatment strategies for HCC. Our research not only demonstrates the therapeutic application of BBR in HCC progression but also, for the first time, elucidates the molecular mechanism by which BBR exerts its antitumor effects through remodeling the tumor immune microenvironment, specifically by inhibiting M2 polarization of macrophages. Furthermore, our experimental results highlight the synergistic effects of combining BBR with anti-PD-L1 antibody therapy, providing a compelling rationale for this combination strategy. Additionally, future investigations should focus on delineating BBR’s regulatory effects on other immune cell components within the tumor microenvironment - particularly neutrophils and dendritic cells - to fully elucidate their specific contributions to BBR-mediated anti-tumor immunity.

Our findings open new avenues for treating HCC and other solid tumors. Firstly, BBR as a natural compound offers advantages of high safety and low cost. Secondly, the synergistic effects between BBR and existing immunotherapies provide novel insights for enhancing clinical efficacy, potentially improving cancer treatment outcomes and warranting further investigation in clinical settings.

## Data Availability

The datasets presented in this study can be found in online repositories. The names of the repository/repositories and accession number(s) can be found in the article/supplementary material.

## Glossary

Arg1: Arginase-1
BCA: Bicinchoninic Acid Assay
BBR: Berberine
BMDMs: Bone marrow-derived macrophages
CAFs: Cancer-associated fibroblasts
Chil3: Chitinase-3-like protein 3
CETSA: Cellular Thermal Shift Assay
COVID-19: Corona Virus Disease 2019
DARTS: Drug Affinity Responsive Target Stability
DCs: Dendritic cells
GAPDH: Glyceraldehyde-3-phosphate dehydrogenase
GBM: Glioblastoma multiforme
GEO: Gene Expression Omnibus
HCC: Hepatocellular carcinoma
ICIs: Immune checkpoint inhibitors
IL-4: Interleukin-4
IL-4R: Interleukin-4 receptor
IL-10: Interleukin-10
i.g.: intragastric administration
i.p.: intraperitoneal injection
JAK1: Janus kinase 1
M-CSF: Macrophage-Colony Stimulating Factor
MDSC: Myeloid-derived suppressor cells
Mrc1: Mannose receptor C-type 1
PBS: Phosphate Buffered Saline
PCA: Principal Component Analysis
PCs: Principal components
PD-1: Programmed death 1
PDGF: Platelet-derived growth factor
PD-L1: Programmed Cell Death Ligand 1
PIC: Piceatannol
qPCR: Quantitative real time polymerase chain reaction
RIPA: Radioimmunoprecipitation assay
scRNA-seq: single-cell RNA sequencing
STAT6: Signal Transducer And Activator Of Transcription 6
TACE: Transcatheter arterial chemoembolization
TAM: Tumor-associated macrophages
TGF-β1: Transforming growth factor beta-1
TGFBR1: Transforming growth factor beta receptor 1
TIME: Tumor immune microenvironment
UMAP: Uniform Manifold Approximation and Projection
VEGF: Vascular endothelial growth factor.
